# Impact of timing, age and parity on clinicopathologic and prognostic factors in pregnancy-associated breast cancer

**DOI:** 10.1007/s12672-026-04712-y

**Published:** 2026-03-17

**Authors:** M. A. Elgendy, N. Abdalkader, A. Elsayed, I. Moaz, A. M. Helal

**Affiliations:** 1 Newgiza University, Cairo, Egypt; 2 National Liver Institute, Menoufia University, Menoufia, Egypt; 3 National Cancer Institute, Cairo University, Cairo, Egypt

## Abstract

This study explores the pathological, clinical, and prognostic characteristics of pregnancy-associated breast cancer (PABC). Our findings challenge specific biological models by revealing no significant differences in local nodal involvement, lymphovascular invasion, pathological type, or hormone receptor status between the pregnancy and postpartum groups. Additionally, recurrence-free survival results revealed worse outcomes in the postpartum group compared to the pregnancy group; however, this disparity did not reach statistical significance. These results align with existing evidence but contradict findings from biological models.

## Introduction

Pregnancy involves significant hormonal and immunological changes. The precise impact of gestational hormones on breast cancer is not fully established [1]. Recent reports indicate that pregnancy has a dual effect on cancer, as it is associated with an increased risk of breast cancer in the years following pregnancy, followed by a long-term protective effect [2]. Pregnancy-associated breast cancer (PABC) is commonly defined as breast cancer diagnosed during pregnancy or one year postpartum [3]. Recent studies suggest differentiation between breast cancer diagnosed during pregnancy (PrBC) and during the postpartum period (PPBC), as they show distinct pathological characteristics and prognostic outcomes [4, 5].

The most common cancer diagnosed in pregnancy is breast cancer, with a prevalence of 1 in 3000 pregnancies [6]. The terms PrBC and PABC have been used interchangeably in epidemiology reports, while the former describes cancer diagnosed during pregnancy and the latter describes cancer diagnosed during pregnancy and 1 year postpartum [7]. Recent reports that separate PrBC and PPBC estimate PrBC prevalence to be 4% and PPBC to be 35–55% of breast cancer cases in women younger than 45 years [5, 8, 9].

PABC is associated with a higher risk of death (pooled hazard ratio (pHR): 1.44 compared to non-PABC controls and typically presents with aggressive profiles (higher stage, size, and lymph node metastases) [2, 10]. However, when further examined, it was found that PrBC shows no distinguishable difference in prognosis, while PPBC showed a poorer prognosis with worse survival rates and with over double the risk of metastasis when compared to non-PABC controls [4, 5, 11]. This difference in outcome prompted the need for differentiation between PrBC and PPBC. It is worth noting that PABC is often diagnosed at later stages due to multiple factors: women in reproductive years do not undergo routine screening, its clinical signs are dismissed as gestational changes, invasive investigational procedures are not preferred in pregnant or lactating women, and gestational changes to breast tissue make detection and mammogram interpretation more difficult [2, 12, 13].

It’s thought that pregnancy has a cancer-promoting effect through hormonal changes in estrogen, progesterone, and Insulin-like Growth Factor 1 (IGF-1), along with pregnancy-induced immune suppression, which could promote growth to already initiated cells [2]. After delivery and during weaning, involution occurs, during which the breast tissue restructures to return to its pre-pregnancy state [5]. Involution is characterized by the cell death of 50–80% of the mammary epithelium, accompanied by remodeling—a process similar to wound healing and inflammation, both of which are associated with tumor growth and metastasis [5, 9]. It’s hypothesized that involution increases the metastatic potential of already initiated cells, which could explain the poor prognosis [5]. PABC tumors are primarily triple-negative or HER2-amplified invasive ductal carcinomas, presenting with higher grades, larger sizes, and higher rates of lymph node involvement than their non-PABC counterparts [7, 14].

The primary aim of this study is to investigate the differences in clinicopathological features and recurrence-free survival between PrBC and PPBC cases. This study’s secondary aim is to examine the differences in PABC cases when grouped by age and gravidity.

## Methodology

### Study design

This is a retrospective cohort study aimed at investigating the clinicopathological differences between postpartum breast cancer (PPBC) and pregnancy breast cancer (PrBC). The study’s goal was to include all patients diagnosed with PABC, recorded in Baheya Hospital’s electronic medical records (EMR) between 2015 and 2023. PABC was defined as breast cancer occurring during pregnancy or within the first year postpartum. The timeframe for diagnosis was determined based on the onset of symptoms, or, if unavailable, the date of the biopsy. Excluded patients were those with ipsilateral recurrent breast cancer during pregnancy or contralateral recurrent breast cancer with the same biological profile.

### Data collection process

The initial sampling pool comprised approximately 15,000 patients from Baheya Hospital’s EMR system. A keyword search (e.g., “breast cancer,” “pregnancy,” “lactation”; complete list in Supplement 1) excluding postmenopausal patients yielded 592 records. Of these, 532 did not meet PABC criteria (diagnosis during pregnancy or ≤ 1 year postpartum). Among 60 potential cases, 24 lacked data on pregnancy timing and could not be classified as PrBC or PPBC. The final cohort included 36 cases, which reflects the rarity of PABC (~ 1 in 3,000 pregnancies) [6, 7].

**Fig. 1:**
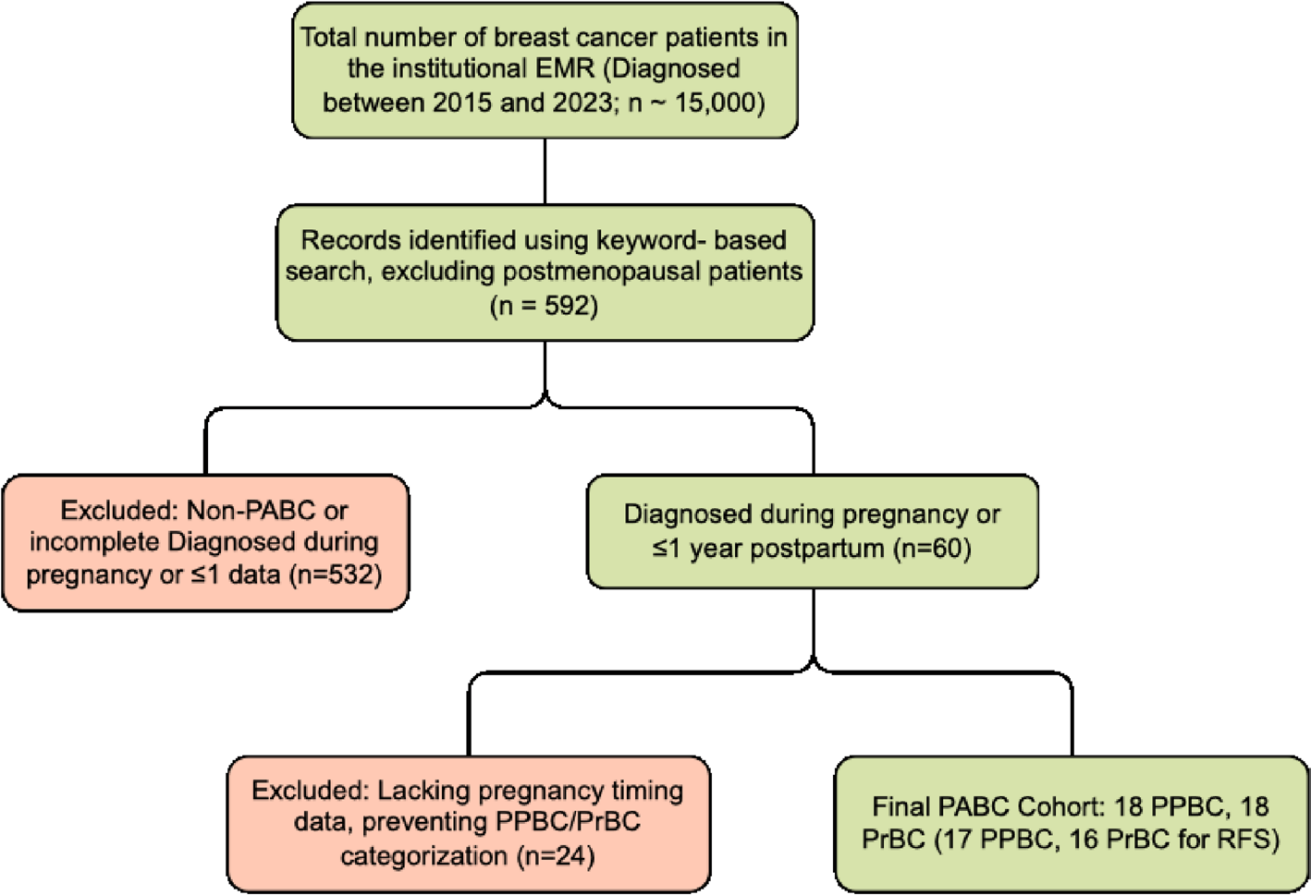
PABC patient selection flowchart

### Statistical analysis

Medians (with interquartile ranges, IQR) were used to describe continuous variables, and frequencies (%) were used for categorical variables. The sample was divided into subgroups based on the type of PABC (PrBC and PPBC), age (above and below 35 years), and gravidity (above and below two pregnancies). Subgroups’ clinicopathologic characteristics were compared using the Mann-Whitney U test for continuous variables and Fisher’s exact test and Chi-square for categorical variables. Recurrence-Free Survival (RFS) was defined as the period from the surgery date to the date of clinically diagnosed recurrence or the last known follow-up. Two-year recurrence-free survival curves were computed using the Kaplan-Meier method and compared using the Log-rank test. R statistical package version 4.4.1 was used for descriptive statistics and subgroup comparisons. GraphPad Prism version 10 was used for the analysis of recurrence-free survival.

### Ethical considerations

All methods were carried out in accordance with relevant guidelines and regulations. The Baheya Hospital’s Institutional Review Board granted ethical approval. All data used in this study were obtained from Baheya’s electronic medical records, for which healthcare providers obtained prior informed consent for the secondary use of health information in research.

## Results

The study cohort comprised 36 patients, half of whom were in the postpartum group (*n* = 18) and half in the pregnancy group (*n* = 18). The median age for all participants was 34.5 years (IQR: 32–39). Most of the cohort had a negative family history of breast cancer (20, 62.5%) and a history of over two pregnancies (21, 65.63%). Almost half of the postpartum group (7, 46.67%) had a positive family history compared to only a third in the pregnancy group (5, 29.4%). However, that difference was not significant (*p* = 0.314). Over half of the pregnancy group was diagnosed in the first trimester (7, 46.67%) and the second trimester (2, 12.33%). Most of the postpartum group was diagnosed between 6 and 12 months postpartum (11, 61.1%). The median time from symptom onset to first-line treatment differed between the two groups, ranging from 118 days for the postpartum group (IQR, 59–330 days) to 184 days for the pregnancy group (IQR, 162–233 days). However, this difference wasn’t significant (*p* = 0.136) (Table 1).

### Pathological features

Most participants had grade 2 (23, 63.89%), unicentric tumors (28, 77.78%) categorized as invasive ductal carcinoma (IDC) (86.11%) with a luminal subtype receptor profile (29, 80.5%). Around a third had peri-tumoral lymphovascular invasion (PT-LVI) in the biopsy (10, 29.41%). DCIS was absent in half of the biopsies (18, 54.55%), with approximately a third ranging from intermediate grade (7, 21.21%) to high-grade DCIS (4, 12.12%).

The two groups had overall comparable distributions regarding pathological features with no significant differences. However, the groups differed significantly in terms of tumor centricity (*p* = 0.0029). Only half of the postpartum group had unicentric tumors (10, 55.56%), while the other half had multicentric tumors (8, 44.44%). In contrast, all participants in the pregnancy group had unicentric tumors (Table 1).

**Table 1:** Demographic and pathological characteristics

Feature	All (*n* = 36)	Postpartum (*n* = 18)	Pregnancy (*n* = 18)	*p*
*Median Age*,* IQR*	34.5 (32–39)	34 (29.5–38.5)	35.5 (33–39)	0.3414
*Age Group*				0.5023
Above 35	16 (44.44%)	7 (38.89%)	9 (50.00%)	
Equal to or under 35	20 (55.56%)	11 (61.11%)	9 (50.00%)	
*Gravida*				> 0.9999
1	3 (9.38%)	2 (11.11%)	1 (7.14%)	
2	8 (25%)	4 (22.22%)	4 (28.57%)	
> 2	21 (65.63%)	12 (66.67%)	9 (64.29%)	
*FH of BC*				0.314
Positive	12 (37.5%)	7 (46.67%)	5 (29.4%)	
Negative	20 (62.5%)	8 (53.33%)	12 (70.6%)	
*Median Time to Treatment (days)*	180 (112–306)	118 (59–330)	184 (162–233)	0.1362
*Timing of Diagnosis*				
First Trimester	7 (21.21%)		7 (46.67%)	
Second Trimester	2 (6.061%)		2 (13.33%)	
Third Trimester	6 (18.18%)		6 (40.00%)	
Within 6 months	7 (21.21%)	7 (38.89%)		
Between 6–12 months	11 (33.33%)	11 (61.11%)		
*Pathological Type*				0.4039
IDC	31 (86.11%)	14 (77.78%)	17 (94.44%)	
ILC	2 (5.56%)	2 (11.11%)		
Other	3 (8.33%)	2 (11.11%)	1 (5.56%)	
*Centricity*				0.0029
Unicentric	28 (77.78%)	10 (55.56%)	18 (100%)	
Multicentric	8 (22.22%)	8 (44.44%)		
*Tumor Grade*				0.5583
1	2(5.56%)	2 (11.11%)		
2	23(63.89%)	11 (61.11%)	12 (66.67%)	
3	11(30.56%)	5 (27.78%)	6 (33.33%)	
*Receptor Status*				0.4872
HR-positive/HER2-negative	29 (80.5%)	13 (72.22%)	16 (89.89%)	
HER2 positive	2 (5.5%)	2 (11.11%)		
TNBC	5 (13.8%)	3 (16.67%)	2 (11.11%)	
*PT-LVI (Biopsy)*				0.8245
Present	10 (29.41%)	5 (27.78%)	5 (27.78%)	
Absent	24 (70.59%)	13 (72.22%)	11 (61.11%)	
*PNI (Specimen)*				0.2273
Present	3 (8.33%)	3 (17.65%)		
Absent	30 (83.33%)	14 (82.35%)	16 (100.00%)	
*DCIS (biopsy)*				0.2152
Absent	18 (54.55%)	10 (55.56%)	8 (53.33%)	
Low grade	4 (12.12%)	4 (22.22%)		
Intermediate grade	7 (21.21%)	3 (16.67%)	4 (26.67%)	
High grade	4 (12.12%)	1 (5.56%)	3 (20.00%)	

*FH* Family history,* BC* Breast cancer,* IDC* Invasive ductal carcinoma,* ILC* Invasive lobular carcinoma,* Centricity* defined as the presence of multiple tumor foci in different breast quadrants,* HR* Hormone,* TNBC* Triple-negative breast cancer,* PT-LVI* Peri-tumoral lymph vascular invasion,* PNI (Specimen)* Perineural invasion,* DCIS (biopsy)* Ductal carcinoma in situ

### Clinical and prognostic stages

Clinical tumor (cT) and node (cN) stages showed no significant differences between groups. Regarding cT staging, over half (12, 33.3%) ranged from T3 to T4 (9, 25%). Around half were N1 (17, 47.22%) while a quarter was N3 (9, 25%). Few cases had metastasis at presentation, with only 8.33% of patients having M1 disease (*p* > 0.9999). It’s worth noting that the postpartum group had a higher percentage in T3 (7, 38.89%) while the pregnancy group had a higher percentage in T4 (6, 33.33%). Additionally, most of the postpartum group were N1 (12, 66.67%) compared to only a third in the pregnancy group (5, 27.78%). Most patients were classified as stage 3 in the AJCC anatomic staging (25, 69.7%), and nearly half were classified as stage 3 in the AJCC prognostic staging (16, 44.4%) (Table 2).

**Table 2:** Tumor grading and staging

Feature	All (*n* = 36)	Postpartum (*n* = 18)	Pregnancy (*n* = 18)	*p*
*Clinical TNM Staging*				
*cT*				0.7391
T1	6 (16.67%)	3 (16.67%)	3 (16.67%)	
T2	8 (22.22%)	4 (22.22%)	4 (22.22%)	
T3	12 (33.33%)	7 (38.89%)	5 (27.78%)	
T4	9 (25.00%)	3 (16.67%)	6 (33.33%)	
Tx	1 (2.78%)	1 (5.56%)		
*cN*				0.1007
N0	5 (13.89%)	1 (5.56%)	4 (22.22%)	
N1	17 (47.22%)	12 (66.67%)	5 (27.78%)	
N2	5 (13.89%)	1 (5.56%)	4 (22.22%)	
N3	9 (25.00%)	4 (22.22%)	5 (27.78%)	
*M at presentation*				> 0.9999
M0	33 (91.67%)	17 (94.44%)	16 (88.89%)	
M1	3 (8.33%)	1 (5.56%)	2 (11.11%)	
*AJCC anatomic stage*				0.6022
1	3 (8.33%)	1 (5.56%)	2 (11.11%)	
2	5 (13.89%)	4 (22.2%)	1 (5.6%)	
3	25 (69.4%)	12 (66.7%)	13 (72.2%)	
4	3 (8.33%)	1 (5.6%)	2 (11.1%)	
*AJCC prognostic stage*				> 0.9999
0	1 (2.78%)	1 (5.56%)		
1	6 (16.67%)	3 (16.67%)	3 (16.67%)	
2	10 (27.78%)	4 (22.22%)	5 (27.78%)	
3	16 (44.44%)	9 (50.00%)	8 (44.44%)	
4	3 (8.33%)	1 (5.56%)	2 (11.11%)	

### Recurrence-free survival in pregnancy and postpartum patients

The total number of recurrence events observed in this study was 5, with 4 events occurring in the postpartum breast cancer (PPBC) group and 1 event in the pregnancy breast cancer (PrBC) group.

**Fig. 2:**
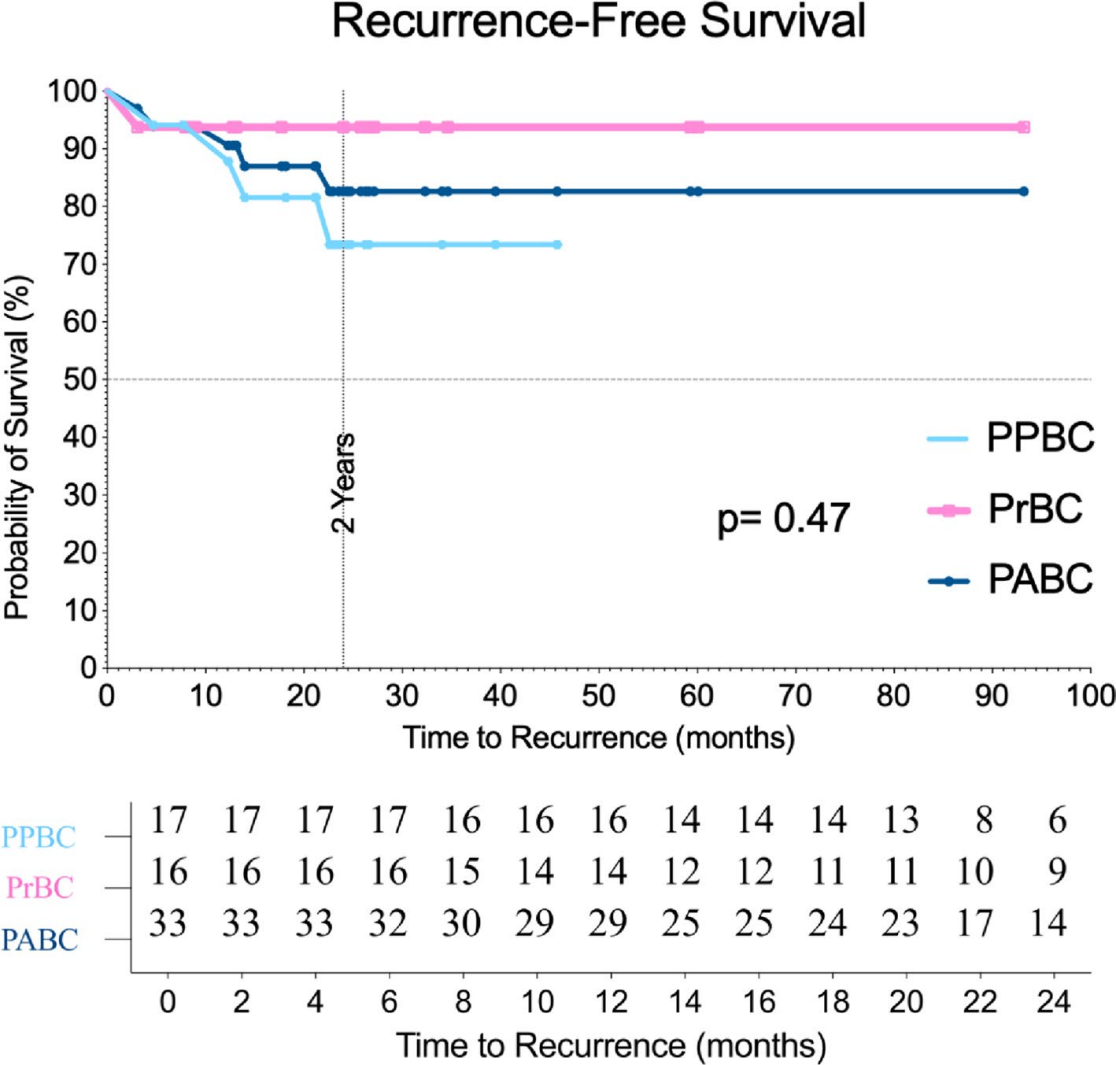
Recurrence-free survival

RFS rate at 2 years for the entire cohort was 82.61% (95% CI: 69.71%−95.51%). When stratified by type of PABC, the RFS for the postpartum group was 73.41% (95% CI: 52.38−94.44%), while the RFS for the pregnancy group was 93.75% (95% CI: 81.93% − 100.00%) (Fig. 2).

### Grouping according to age

When grouped according to age, the > 35 group had 16 participants, and the ≤ 35 group had 20 participants. The two groups significantly differed in terms of specimen PT-LVI, as most of the > 35 group (9, 60%) had present lymph-vascular invasion in the tumor specimen, compared to almost a quarter (4, 22.2%) in the ≤ 35 group (*p* = 0.0377). Specimen DCIS grades also differed significantly, as around half of the > 35 group (8, 53.33%) showed an intermediate grade, versus most of the ≤ 35 group (12, 70.59%) being absent (*p =* 0.0002). It’s worth noting that the two groups differed in cN as the majority of the ≤ 35 group (12, 60%) was N1, while a third of the > 35 group (5, 31.25%) was N1. However, this difference was not significant (Table 3).

**Table 3:** Pathological features and grading according to age groups

Feature	Age > 35 (*n* = 16)	Age ≤ 35 (*n* = 20)	*p*
*Pathological Type*			> 0.9999
IDC	14 (87.50%)	17 (85.00%)	
Others	2 (12.50%)	3 (15.00%)	
*ER Status*			0.2571
Negative	2 (12.50%)	6 (30.00%)	
Positive	14 (87.50%)	14 (70.00%)	
*PR Status*			> 0.9999
Negative	3 (18.75%)	3 (15.00%)	
Positive	13 (81.25%)	17 (85.00%)	
*HER2/neu Status*			> 0.9999
Amplified	1 (6.25%)	1 (5.00%)	
Non-amplified	15 (93.75%)	19 (95.00%)	
*Centricity*			0.4217
Unicentric	11 (68.75%)	17 (85.00%)	
Multicentric	5 (31.25%)	3 (15.00%)	
*PT-LVI (Specimen)*			0.0377
Absent	6 (40.00%)	14 (77.78%)	
Present	9 (60.00%)	4 (22.22%)	
*DCIS (specimen)*			0.0002
Absent	2 (13.33%)	12 (70.59%)	
Low grade		3 (17.65%)	
Intermediate grade	8 (53.33%)	2 (11.76%)	
High grade	4 (26.67%)		
Present – grade indetermined	1 (6.67%)		
*TNM Staging*			
*cT*	3 (18.75%)	3 (15.79%)	0.7992
T1	4 (25.00%)	4 (21.05%)	
T2	4 (25.00%)	8 (42.11%)	
T3	5 (31.25%)	4 (21.05%)	
T4			
*cN*			0.3829
N0	3 (18.75%)	2 (10.00%)	
N1	5 (31.25%)	12 (60.00%)	
N2	3 (18.75%)	2 (10.00%)	
N3	5 (31.25%)	4 (20.00%)	

*ER* Estrogen,* PR* Progesterone,* HER2* Human epidermal growth factor 2,* Centricity* defined as the presence of multiple tumor foci in different breast quadrants,* PT-LVI* Peri-tumoral lymph vascular invasion,* DCIS (biopsy)* Ductal carcinoma in situ

### Grouping according to gravidity

When grouped according to gravidity, the > 2 gravida group had 21 patients, and the ≤ 2 gravida group had 11 patients. Specimen PT-LVI differed between the two groups (*p* = 0.0575). Specimen PT-LVI was present in approximately half of the > 2 gravida group (11, 57.89%), while it was mostly absent in the ≤ 2 gravida group (9, 81.82%). The two groups significantly differed in PR receptor status as almost all the > 2 gravida group (20, 95.24%) were positive compared to only 63.64% of the ≤ 2 gravida group (Table 4).

**Table 4:** Pathological features when grouped according to gravidity

Feature	Gravida > 2 (*n* = 21)	Gravida 1 and 2 (*n* = 11)	*p*
*Median Age*,* IQR (years)*	36 (36–40.5.5)	33 (33–37)	0.1
*Type of PABC*			0.8881
PPBC	12 (57.14%)	6 (54.55%)	
PrBC	9 (42.86%)	5 (45.45%)	
*Centricity*			0.6808
Centric	15 (71.43%)	9 (81.82%)	
Multicentric	6 (28.57%)	2 (18.18%)	
*PT-LVI (Specimen)*			0.0575
Absent	8 (42.11%)	9 (81.82%)	
Present	11 (57.89%)	2 (18.18%)	
*DCIS (Specimen)*			0.2403
Absent	8 (42.11%)	6 (60.00%)	
High grade	1 (5.26%)	1 (10.00%)	
Intermediate grade	8 (42.11%)	1 (10.00%)	
Low grade	1 (5.26%)	2 (20.00%)	
Present - grade indetermined	1 (5.26%)	0 (0.00%)	
*ER Status*			0.0877
Negative	3 (14.29%)	5 (45.45%)	
Positive	18 (85.71%)	6 (54.55%)	
*PR Status*			0.0367
Negative	1 (4.76%)	4 (36.36%)	
Positive	20 (95.24%)	7 (63.64%)	
*HER2 Status*			> 0.9999
Amplified	1 (4.76%)	1 (9.09%)	
Non-amplified	20 (95.24%)	10 (90.91%)	

*IQR* Interquartile range, PABC: Pregnancy-associated breast cancer,* PPBC* Postpartum breast cancer,* PrBC* Pregnancy breast cancer,* Centricity* defined as the presence of multiple tumor foci in different breast quadrants,* PT-LVI* Peri-tumoral lymph vascular invasion,* DCIS (biopsy)* Ductal carcinoma in situ,* ER* Estrogen,* PR* Progesterone,* HER2* Human epidermal growth factor 2

### Neoadjuvant chemotherapy outcomes

In the cohort, 26 patients received neoadjuvant chemotherapy (NACT). Around a third of patients were staged as ypT2 (11, 30.56%) and ypN1 (8, 29.63%). Around half of the cohort was classified as RCB-III (12, 48%). When grouped according to age, ypN showed a significant difference between the two groups (*p* = 0.0139), as over a third of the > 35 group was ypN2 (3, 37.5%), while for the ≤ 35 group, it was ypN1 (7, 38.89%). Residual viable tumor differs between groups when grouped according to the type of PABC and gravidity. However, it was comparable when grouped according to age. When grouped according to PABC type, the postpartum group had 37.5% residual viable tumor compared to 67.5% in the pregnancy group. According to gravidity grouping, the > 2 gravida group had 62.5% residual viable tumor, while it was 37% for the ≤ 2 gravida group (Tables 5, 6, and 7).

**Table 5:** NACT outcomes when grouped according to the type of PABC

Neoadjuvant pathology	All (*n* = 26)		Postpartum (*n* = 13)		Pregnancy (*n* = 13)	*p*
*ypT*						0.8999
ypTis	1 (2.78%)				1 (7.69%)	
ypT0	5 (13.89%)		3 (23.08%)		2 (15.38%)	
ypT1	6 (16.67%)		4 (30.77%)		2 (15.38%)	
ypT2	11 (30.56%)		4 (30.77%)		6 (46.15%)	
ypT3	2 (5.56%)		1 (7.69%)		1 (7.69%)	
ypT4	2 (5.56%)		1 (7.69%)		1 (7.69%)	
*ypN*						0.6022
ypN0	10 (37.04%)		4 (30.77%)		6 (46.15%)	
ypN1	8 (29.63%)		5 (38.46%)		3 (23.08%)	
ypN2	5 (18.52%)		1 (7.69%)		3 (23.08%)	
ypN3	4 (14.81%)		3 (23.08%)		1 (7.69%)	
*Median Residual Viable Tumor*,* IQR*	50% (10–78.8.8%)		37.5% (21.2–66.3%)		67.5% (7.5–90%)	0.5963
*RCB Class*						0.3226
RCB-0	5 (20%)		3 (23.08%)		2 (15.38%)	
RCB-I	1 (4%)				1 (7.69%)	
RCB-II	7 (28%)		2 (15.38%)		5 (38.46%)	
RCB-III	12 (48%)		8 (61.54%)		4 (30.77%)	

*IQR* Interquartile range,* RCB* Residual cancer burden score,* Residual Viable Tumor* the proportion of tumor cells that remain alive and capable of growth and proliferation following neoadjuvant chemotherapy

**Table 6:** NACT outcomes when grouped according to age groups

Post-NACT pathology	Age > 35 (*n* = 8)	Age ≤ 35 (*n* = 18)	*p*
*ypT*			0.7698
ypTis		1 (5.56%)	
ypT0	2 (25%)	3 (16.67%)	
ypT1	1 (12.5%)	5 (27.78%)	
ypT2	4 (50%)	6 (33.33%)	
ypT3	1 (12.5%)	1 (5.56%)	
ypT4		2 (11.11%)	
*ypN*			0.0139
ypN0	1 (12.5%)	9 (50.00%)	
ypN1	1 (12.5%)	7 (38.89%)	
ypN2	3 (37.5%)	1 (5.56%)	
ypN3	3 (37.5%)	1 (5.56%)	
*Median Residual Viable Tumor*,* IQR*	52.5% (3.7–90%)	47.5% (10–73.7.7%)	0.8448
*RCB Class*			0.3002
RCB-0	1 (12.5%)	4 (23.53%)	
RCB-I	1 (12.5%)	0 (0.00%)	
RCB-II	1 (12.5%)	6 (35.29%)	
RCB-III	5 (62.50%)	7 (41.18%)	

*IQR* Interquartile range,* RCB* Residual cancer burden score,* Residual Viable Tumor* the proportion of tumor cells that remain alive and capable of growth and proliferation following neoadjuvant chemotherapy

**Table 7:** NACT outcomes when grouped according to type of gravidity

Feature	Gravida > 2 (*n* = 14)	Gravida 1 and 2 (*n* = 9)	*p*
*ypT*			> 0.9999
ypTis	1 (7.14%)	0 (0.00%)	
ypT0	2 (14.29%)	2 (22.22%)	
ypT1	4 (28.57%)	2 (22.22%)	
ypT2	5 (35.71%)	4 (44.44%)	
ypT3	1 (7.14%)	0 (0.00%)	
ypT4	1 (7.14%)	1 (11.11%)	
*ypN*			0.9813
ypN0	5 (35.71%)	4 (44.44%)	
ypN1	3 (21.43%)	3 (33.33%)	
ypN2	3 (21.43%)	1 (11.11%)	
ypN3	3 (21.43%)	1 (11.11%)	
*Median Residual Viable Tumor*,* IQR*	62.5% (15–90%)	37% (7.5–78.7%)	0.488
*RCB class*			> 0.9999
RCB-0	3 (21.43%)	2 (22.22%)	
RCB-II	4 (28.57%)	2 (22.22%)	
RCB-III	7 (50.00%)	5 (55.56%)	

*IQR* Interquartile range,* RCB* Residual cancer burden score,* Residual Viable Tumor* the proportion of tumor cells that remain alive and capable of growth and proliferation following neoadjuvant chemotherapy

## Discussion

This study explores the pathological, clinical, and prognostic characteristics of pregnancy-associated breast cancer (PABC), with particular emphasis on comparing postpartum breast cancer (PPBC) and pregnancy breast cancer (PrBC). Additional analyses were conducted when cases were categorized by age and parity to understand subgroup differences better.

Our results revealed no significant differences in local nodal involvement (cN), lymphovascular invasion (LVI), pathological type, or hormone receptor status between PPBC and PrBC, despite animal model studies suggesting a poorer prognosis in PPBC due to increased lymphovascular density. Involution in the postpartum breast has been shown to increase lymphovascular density and metastatic potential through a prolymphangiogenic process similar to inflammation and wound healing [8, 15]; however, these features were not clearly evident in our cohort. Instead, only around one-third of PPBC cases demonstrated peri-tumoral LVI, and most had no distant metastasis at presentation. These discrepancies highlight the challenges of reconciling biologic models with clinical findings and underscore the importance of standardized definitions of PPBC across studies.

A significant finding in our study was the difference in tumor centricity: PPBC cases were more frequently multicentric (8, 44.44%) than PrBC cases. Multicentricity has been associated with increased nodal metastasis and worse overall survival outcomes, suggesting that PPBC has poorer survival outcomes and possibly different biological behavior compared to PrBC [16–18]. Thus, multicentricity/multifocality can be considered an early prognostic marker for PPBC, granting a different clinical management course.

Due to data limitations, a 2-year RFS comparison between PPBC and PrBC was conducted instead of a longer RFS. Our cohort demonstrated a numerically lower 2-year RFS in the postpartum group compared with the pregnancy group (73.4% vs. 93.8%), although the difference was not statistically significant. These short-term results align with previous studies that used a narrow definition of PPBC (≤ 1 year postpartum) and reported no significant differences in early outcomes [19, 20]. In contrast, studies adopting broader definitions of PPBC (5–10 years postpartum) consistently demonstrated worse long-term survival and higher recurrence risks compared with nulliparous women, particularly in ER-positive disease [21, 22]. Taken together, our findings suggest that the adverse prognosis of PPBC may not be fully captured when using a narrow postpartum window and short follow-up.

Stratification of the cohort revealed clinically relevant differences: patients > 35 years exhibited higher levels of nodal involvement, while women with more than two pregnancies demonstrated significantly higher rates of LVI. These findings reinforce prior evidence that reproductive history and maternal age influence the biology and prognosis of breast cancer and highlight the need for larger, stratified studies that adjust for pre-existing risk factors.

The prominent limitation of our study is the small sample size, which limits the power of statistical analyses conducted, particularly when the cohort is divided into smaller subgroups. A post-hoc power analysis for the RFS (PPBC vs. PrBC, *p* = 0.47) was conducted, and it showed low power (40%, HR ≈ 4.2, *n* = 17 in PPBC and *n* = 16 in PrBC) due to only five events, reflecting the limited sample size. This limitation means that negative results should be interpreted with caution, as the limited power increases the likelihood of false negatives. The difficulty in obtaining a sufficiently large sample size for a well-powered study appears to be a common issue among similar studies. A study, which defined PPBC as breast cancer diagnosed up to five years postpartum, included patients from two hospitals in Colorado, one being a cancer center and the other a university hospital, over the span of 30 years managed to recruit 24 PrBC and 136 PPBC patients only [9], another study where PPBC defined as 1 year postpartum, recruited patients from a university hospital from 2009 to 2021 recruited only 34 PABC patients [10]. Collecting a sufficiently large sample remains the biggest obstacle in conducting a well-powered study; it’s partly due to the underreporting of PABC, as well as the rarity of the condition itself. We recommend that future research focus on compiling nationwide patient data and conducting meta-analyses.

Our study thus presents an opportunity to generate hypotheses regarding the possible existence of biological and clinical differences between PrBC and PPBC, particularly when PPBC includes patients up to 5 years postpartum, and given the higher frequency of multicentric disease in PPBC—which is associated with worse outcomes—prospective studies are needed to assess whether PPBC patients would benefit from more aggressive initial treatment strategies.

## Data Availability

Due to privacy and ethical restrictions, the datasets are not publicly available. De-identified data may be made available upon reasonable request and with appropriate institutional approvals.
